# Trajectories of pain intensity, pain catastrophizing, and pain interference in the perinatal and postpartum period

**DOI:** 10.1097/PR9.0000000000001137

**Published:** 2024-01-07

**Authors:** Jenna Jessa, Lianne Tomfohr-Madsen, Ashley Dhillon, Andrew Walker, Melanie Noel, Ivan Sedov, Jillian Vinall Miller

**Affiliations:** a Department of Anesthesiology, Perioperative & Pain Medicine, Cumming School of Medicine, University of Calgary, Calgary, AB, Canada; b Vi Riddell Children's Pain & Rehabilitation Centre, Alberta Children's Hospital, Calgary, AB, Canada; c The Mathison Centre for Mental Health Research & Education, Hotchkiss Brain Institute, Calgary, AB, Canada; d Child Brain & Mental Health Program, Alberta Children's Hospital Research Institute, Calgary, AB, Canada; e Owerko Centre, Alberta Children's Hospital Research Institute, Calgary, AB, Canada; f O'Brien Institute for Public Health, University of Calgary, Calgary, AB, Canada; g Faculty of Education: Educational and Counselling Psychology, and Special Education, University of British Columbia, Vancouver, BC, Canada; h Department of Psychology, University of Toronto, Toronto, ON, Canada; i Alberta Health Services, Calgary, AB, Canada; j Department of Psychology, Cumming School of Medicine, University of Calgary, Calgary, AB, Canada

**Keywords:** Gestation, Maternal, Pain assessment, Pregnancy, Prenatal, Postnatal

## Abstract

**Introduction:**

Chronic pain (pain >3 months) is a growing epidemic. Normal pregnancy may give rise to recurrent and sometimes constant pain for women. Women with worse pain symptoms are more likely to report symptoms of anxiety, depression, and/or insomnia during the perinatal period, which may impact labor and delivery outcomes. We examined the relationship between demographic and psychological predictors of pain throughout pregnancy and into the postpartum.

**Objectives:**

To examine trajectories of pain intensity, pain catastrophizing, and pain interference during pregnancy and the early postpartum, and associated sociodemographic predictors of trajectory membership.

**Methods:**

One hundred forty-two pregnant women were assessed at 4 time points for measures of pain intensity, pain catastrophizing, pain interference, and symptoms of insomnia, depression, and generalized anxiety. Women completed the first survey before 20 weeks’ gestation and were reassessed every 10 weeks. Surveys were completed on average at 15 weeks', 25 weeks', and 35 weeks' gestation, and at 6-week postpartum. Using latent class mixed models, trajectory analysis was used to determine trajectories of pain intensity, pain catastrophizing, and pain interference.

**Results:**

A 1-class pain intensity model, 2-class pain catastrophizing model, and 3-class pain interference model were identified. Adaptive lasso and imputation demonstrated model robustness. Individual associations with trajectories included baseline symptoms of anxiety, depression, and insomnia, and pain symptomology.

**Conclusion:**

These findings may help to identify women who are at high risk for experiencing pain symptoms during pregnancy and could aid in developing targeted management strategies to prevent mothers from developing chronic pain during their pregnancy and into the postpartum period.

## Abstract 2

Supplemental Digital Content is Available in the Text.

Maternal trajectories of pain catastrophizing and pain interference in the perinatal period and into the postpartum period are associated with baseline mental health and pain symptomology.

## 1. Introduction

Globally, chronic pain is the leading cause of years lived with disability.^23^ Women are overrepresented as patients with chronic pain, with upward of 70% of patients with chronic pain identifying as women.^16^ Pain experienced during pregnancy presents a unique burden on expecting mothers because it is an ailment often associated with a normal pregnancy. Reports of pain prevalence during pregnancy widely vary in the literature, ranging between 30% and 85% depending on how, when, and what kind of pregnancy-related pain was assessed.^9,29,45^ Pain experienced during pregnancy may also persist chronically to the postpartum in up to 15% of women.^22,36^

Pregnancy-related pain has been associated with disruptions to activities of daily living, including but not limited to physical activity, hygiene, and work-related functioning.^6,14^ This can lead to feelings of dependence and inadequacy, as well as a loss of personal identity, because unmanaged pain may require women to take premature leave from work, affect their sexual relationship with their partner, and/or make it difficult to engage in self-care.^6,14^ Pain interference, or the extent to which pain affects an individual's activities of daily living, is thus an important consideration in understanding pain experience.^2^

Pain during pregnancy has been strongly associated with psychological distress, including pain catastrophizing, which is the tendency to ruminate, magnify, or feel helpless in the presence of pain.^16,33,42^ Women who report greater pain catastrophizing are more likely to report increased pain intensity both predelivery and postdelivery and poorer physical recovery when returning to activities of daily living.^20^ Women with greater pain severity are also more likely to report symptoms of anxiety, depression, and/or insomnia during the perinatal period, which may have implications for their labor and delivery.^48^ Pregnancy-related pain has been identified as a risk factor for longer labor, emergency caesarean section, assisted delivery, and/or increased complications during birth.^7^ The concurrence of anxiety, depression, insomnia, and chronic pain has been associated with preeclampsia, gestational diabetes, and preterm birth.^1,46^ It is thus important to identify women who may suffer from compounded risks of pain to prevent adverse perinatal outcomes.

Longitudinal studies have been conducted to describe pain experiences during pregnancy and to determine when symptoms are likely to first occur or worsen.^5,14,29^ Pain manifests in a broad array of bodily regions, including the truncal regions (lower back, upper back, and pelvic girdle), feet/ankle, knees, hip, wrists/hands, head, and/or neck.^29,36,45^ The constellation of pain, or the type, pattern, and intensity of pain, generally changes for an individual as pregnancy progresses into the postpartum.^17^ Overall, pain appears to progressively worsen as gestational age advances, with increases in both pain locations and severity, before comparatively decreasing in the postpartum.^18^ For example, pain in the lower back and pelvic girdle regions often emerges in the second and third trimesters.^45^ In addition, there are significant increases in reports of hand, neck, low back, hip, knee, and ankle pain during the third trimester.^18^ Higher numbers of pain locations endorsed by women during pregnancy have been associated with increased pain severity. However, pain experiences may change between the prenatal period and postpartum because of childbirth and alterations to daily activities (eg, breastfeeding).^18,24^ This change may be difficult to differentiate if the pain remains localized to the same region such as the truncal or pelvic areas.^18^

]The assumption from longitudinal studies is that most women experience the same pattern of change in pain symptoms over the perinatal period. However, there is evidence that sociodemographic factors (age, body mass index, and ethnicity), parity, amenorrhea, history of hypermobility, and history of pain before pregnancy are risk factors for pain development in pregnancy and postpartum.^34,43,53^ Owing to the variability in pain experiences, it is advantageous to identify specific subgroups of women who follow distinct pain trajectories.^4,18^ Identifying women who are at high risk for experiencing pain symptoms could aid in developing targeted treatment strategies related to pregnancy-related pain.

This exploratory study examined trajectories of pain intensity, pain catastrophizing, and pain interference during pregnancy and early postpartum. It was hypothesized that there would be identifiable subgroups of high and low pain trajectories, which could be associated with the mothers' symptomology at first assessment. Exploration of pain and prospective associations with pain development are needed to better characterize risk factors for persistent pain and adverse perinatal outcomes in this population.

## 2. Methods

### 2.1. Participants

On approval by the University of Calgary Health Research Ethics Board (REB17-0507), 142 pregnant women were recruited from maternity clinics in Western Canada. Participants provided informed consent and answered eligibility screening questions. Inclusion criteria included participants of singleton pregnancy, older than 17 years, and <19 weeks' gestation. Exclusion criteria included the inability to read or answer questions in English and lack of access to a computer. Participants were asked to complete 4 online surveys. A link to the first survey was emailed to participants when they were <20 weeks' gestation. Subsequent surveys were then sent to participants 10 weeks after the completion of the previous survey. All data were collected and stored in REDCap,^25^ a secure, online database.

### 2.2. Measures

#### 2.2.1. Demographic information

Participants self-reported their age, gestational age, ethnicity, highest level of completed education, annual household income, marital status, employment status, prepregnancy height and weight, and parity.

#### 2.2.2. Patient-Reported Outcomes Measurement Information System Pain Intensity short form (3 items)

Participants indicated the intensity of pain that they experienced in the past 7 days, with higher scores indicating a higher degree of pain intensity. This scale has previously shown high internal consistency in community samples (Cronbach alpha = 0.91) and had good internal consistency in this sample (Cronbach alpha = 0.87). A t-score of 50, with a standard deviation of 10, is representative of average pain intensity found in the general American population. Therefore, a t-score of 60 or higher is considered worse than the average. A minimally important difference between time points for nonpain samples has been found to range from 3.5 to 4.5 points.^11^

#### 2.2.3. Pain Catastrophizing Scale (13 items)

Participants reported their catastrophizing in the context of actual or anticipated pain, with higher scores indicating a higher degree of pain catastrophizing. The total Pain Catastrophizing Scale (PCS) score has shown high internal consistency (Cronbach alpha = 0.87–0.93) in clinical samples.^38^ The scale had high internal consistency (Cronbach alpha = 0.94) in the current study sample. A total PCS score of 30 is representative of clinically relevant pain catastrophizing.^51^

#### 2.2.4. Patient-Reported Outcomes Measurement Information System Pain Interference subscale short form (4 items)

Participants were asked to indicate the extent to which pain interfered with daily routines as well as social and physical activities.^2^ Higher scores indicate a higher degree of pain interference. The short form has comparable internal consistency to the full Patient-Reported Outcomes Measurement Information System Pain Interference (Cronbach alpha = 0.96–0.99). There was acceptable internal consistency (Cronbach alpha = 0.72) for the short form in the current sample. A t-score of 50, with a standard deviation of 10, is representative of average pain interference found in the general American population. Therefore, a t-score of above 60 is considered worse than the average. A minimally important difference between time points for nonpain samples has been found to range from 3.5 to 4.5 points.^11^

#### 2.2.5. Insomnia Severity Index (7 items)

Participants indicated the severity of their insomnia symptoms within the last 2 weeks,^35^ with higher scores indicating greater symptom severity. The Insomnia Severity Index (ISI) has demonstrated excellent reliability in both a community sample and a clinical sample (Cronbach alpha = 0.90–0.91). For a community sample, a cut-off score of 10 was identified.^35^ Insomnia Severity Index internal consistency within this study sample was good (Cronbach alpha = 0.87).

#### 2.2.6. Generalized Anxiety Disorder 7-item scale

Participants indicated the degree to which they experienced symptoms of anxiety in the previous 2 weeks. Higher scores represent a higher frequency of anxiety symptoms. The Generalized Anxiety Disorder 7-item (GAD-7) scale demonstrated excellent internal consistency (Cronbach alpha = 0.92).^50^ The optimal, valid clinical cut-off for a pregnant population has been found to be 13.^49^ There was also good internal consistency for this scale within the current sample (Cronbach alpha = 0.86).

#### 2.2.7. Edinburgh Postnatal Depression Scale (10 items)

Participants reported on their symptoms of depressed mood in the past 7 days. Higher scores represent a greater number of depressive symptoms. Satisfactory psychometric properties were found in both postnatal and nonpostnatal samples of mothers for the Edinburgh Postnatal Depression Scale (EPDS).^12^ The optimal, valid clinical cut-off for a pregnant population has been found to be 13.^12^ Good internal consistency for the EPDS was found in the current sample (Cronbach alpha = 0.86).

### 2.3. Statistical analyses

#### 2.3.1. Descriptive analysis

Descriptive analyses were conducted using SPSS version 27.0 statistical software (IBM, Armonk, NY). Continuous demographic variables were tested for normality using the Shapiro–Wilk (*P* < 0.05) and presented as mean (SD) or median [interquartile range]. Categorical variables were presented as frequency (percentage).

#### 2.3.2. Trajectory analysis

Trajectory analyses were conducted using R studio version 1.0 with R statistical software version 4.1.3 (The R Project for Statistical Computing, Vienna, Austria) and SPSS version 27.0.

#### 2.3.3. Trajectory classes and model selection

Trajectory analyses used latent class mixed modeling, which belongs to the same mixture modeling family as growth mixture modeling (GMM), (LCMM 1.7.8 package in R) to model pain intensity, pain catastrophizing, and pain interference trajectories over time and to assign participants to trajectory membership.^41^ For complete details, please refer to Appendix A, http://links.lww.com/PR9/A219.

#### 2.3.4. Sample size estimation

The required sample size for GMM varies depending on numerous factors, including group differences, relative sizes of groups, and reliability of measurements; however, a minimum threshold of 100 participants has been identified.^13,44^ It has been demonstrated that GMM outperforms other clustering methods in identifying heterogeneity in longitudinal trajectories, even when performed on small sample sizes (<500).^31^

#### 2.3.5. Factors associated with trajectory class

On final model selection and assignment of trajectory membership, characteristics of distinct trajectory groups and baseline associations of trajectory membership were determined. Baseline characteristics of each trajectory class were examined using χ^2^ tests for categorical variables and Mann–Whitney U tests or Kruskall–Wallis tests (≥2 trajectories). Variables were nonparametric and thus reported as median [interquartile range]. To identify critical associations of trajectory membership, univariate (2 trajectories) or multinomial (>2 trajectories) logistic models were used. Odds ratios (ORs) and 95% confidence intervals (CIs) were presented to illustrate the clinical impact of each selected variable on individual trajectories.

#### 2.3.6. Lasso and imputation

Multivariable complete case models to predict baseline characteristics associated with trajectory membership were developed using least absolute shrinkage and selection operator (lasso) regression. Adaptive lasso was used for outcomes with 2 trajectories, whereas multinomial lasso was used for outcomes with greater than 2 trajectories (glmnet 4.1–8 package in R).^21^ Missing data imputation was used to complete a sensitivity analysis of the complete case logistic and multinomial multivariable models (MICE 3.16.0 package in R).^8^ Both complete case and imputed case models were presented using odds ratios, 95% CIs, and *P* values. For complete details, please refer to Appendix A, http://links.lww.com/PR9/A219.

## 3. Results

### 3.1. Sociodemographic characteristics

Sociodemographic characteristics of our recruited cohort are presented in Table 1.

**Table 1:** Sociodemographic characteristics of our cohort at baseline (N = 142).

Sociodemographic variables	
Age (y), median [IQR]	31.0 [28.0–35.0]
Prepregnancy body mass index, median [IQR]	23.2 [20.9–26.5]
Ethnicity	
Arab	2 (1.4%)
Asian (East Asian, South Asian, Southeast Asian, or West Asian)	37 (26.1%)
Black (African Canadian, Haitian, or Jamaican)	2 (1.4%)
Hispanic or Latino	4 (2.8%)
Mixed race or none of the above	7 (4.9%)
White (Caucasian)	90 (63.4%)
Household income	
<$10,000–$49,999	21 (14.8%)
$50,000–$99,999	36 (25.4%)
$100,000–$149,999	35 (24.6%)
$150,000–$199,999	21 (14.8%)
$200,000–$249,000	21 (14.8%)
$250,000<	8 (5.6%)
Marital status	
Married/common law	133 (93.7%)
Divorced/separated/single/never married	9 (6.3%)
Employment	
Full time	106 (74.6%)
Part time	14 (9.9%)
Unemployed	6 (4.2%)
Stay-at-home parent	16 (11.3%)
Education	
Less than high school	2 (1.4%)
High school diploma or equivalent	10 (7.0%)
Postsecondary certificate or diploma	29 (20.4%)
Bachelor's degree	54 (38.0%)
Master's degree	37 (26.1%)
Doctorate or other professional degree	10 (7.0%)
Parity	
Primipara	69 (48.6%)

Continuous data presented as median [interquartile range] per finding of nonnormality (Shapiro–Wilk test, *P* < 0.05).

Categorical data presented as frequency (percentage).

IQR, interquartile range; N, number of participants.

### 3.2. Pain symptomology during pregnancy and early postpartum

The prevalence of clinically significant pain intensity as defined by a t-score above 60 was 4.7% (early-pregnancy), 16.4% (mid-pregnancy), 20.6% (late-pregnancy) and 12.9% (postpartum). The prevalence of clinically significant pain catastrophizing, as defined by a PCS score of 30 or above was 2.3% (early-pregnancy), 3.4% (mid-pregnancy), 1.9% (late-pregnancy) and 2.0% (postpartum). The prevalence of clinically significant pain interference, as defined by a t-score above 60 was 13.1% (early-pregnancy), 24.0% (mid-pregnancy), 25.2% (late-pregnancy) and 6.9% (postpartum) (Table 2).

**Table 2:** Descriptive statistics of key variables.

Variable	Time 1		Time 2		Time 3		Time 4	
N	142		127		117		112	
Gestational age (wk), median [IQR]	15.0 [12.0–17.0]		25.0 [23.0–27.0]		35.0 [33.0–37.0]		—	
	Median [IQR]	n (%) above clinical cut-off or general population averages	Median [IQR]	n (%) above clinical cut-off or general population averages	Median [IQR]	n (%) above clinical cut-off or general population averages	Median [IQR]	n (%) above clinical cut-off or general population averages
Pain Catastrophizing Scale (PCS), total	3.0 [0.0–9.0]	3 (2.3%)	3.0 [0.0–9.0]	4 (3.5%)	2.0 [0.0–7.0]	2 (1.9%)	1.0 [0.0–4.0]	2 (2.0%)
Pain Interference (PROMIS), t-score	53.9 [41.6–57.1]	17 (13.0%)	54.8 [49.6–61.2]	28 (24.1%)	54.8 [49.6–61.2]	27 (25.2%)	54.8 [49.6–61.2]	7 (6.9%)
Pain Intensity (PROMIS), t-score	43.5 [40.2–46.3]	6 (4.6%)	43.5 [40.2–47.1]	19 (16.4%)	44.9 [40.2–49.4]	22 (20.6%)	43.5 [40.2–46.3]	13 (12.9%)
Insomnia (ISI), total	7.0 [3.0–11.0]	43 (32.8%)	7.0 [3.8–10.3]	37 (31.9%)	8.0 [4.0–12.0]	42 (39.3%)	5.5 [2.0–9.0]	21 (20.8%)
Generalized Anxiety Disorder 7-item (GAD-7) scale, total	2.0 [1.0–4.0]	3 (2.3%)	2.0 [0.0–5.0]	5 (4.3%)	2.0 [0.0–5.0]	7 (6.5%)	3.0 [0.0–5.0]	5 (5.0%)
Edinburgh Postnatal Depression Scale (EPDS), total	5.0 [3.0–8.0]	11 (8.4%)	6.0 [2.0–9.0]	14 (12.1%)	5.0 [2.0–9.0]	9 (8.4%)	4.0 [1.0–8.0]	7 (6.9%)

Continuous data presented as median [interquartile range] per finding of nonnormality (Shapiro–Wilk test, *P* < 0.05).

Categorical data presented as frequency (percentage).

Clinical cut-offs/scores above population averages: (1) Pain Catastrophizing Scale (PCS): total score above 30. (2) Pain Interference and Pain Intensity (PROMIS): t-scores above 60. (3) Insomnia (ISI): total score above 10. (4) Generalized Anxiety Disorder (GAD-7): total score above 13. (5) Edinburgh Postnatal Depression Scale (EPDS): total score above 13.

IQR, interquartile range; N, number of participants.

### 3.3. Model selection

#### 3.3.1. Pain intensity modeling

After fitting the trajectory models, the summed Akaike information criterion (AIC) and Bayesian information criterion (BIC) indicated model fit did not improve with increasing numbers of classes, such that a single trajectory best fit the data (Supplemental Table S1, http://links.lww.com/PR9/A219).

**Figure 1.**
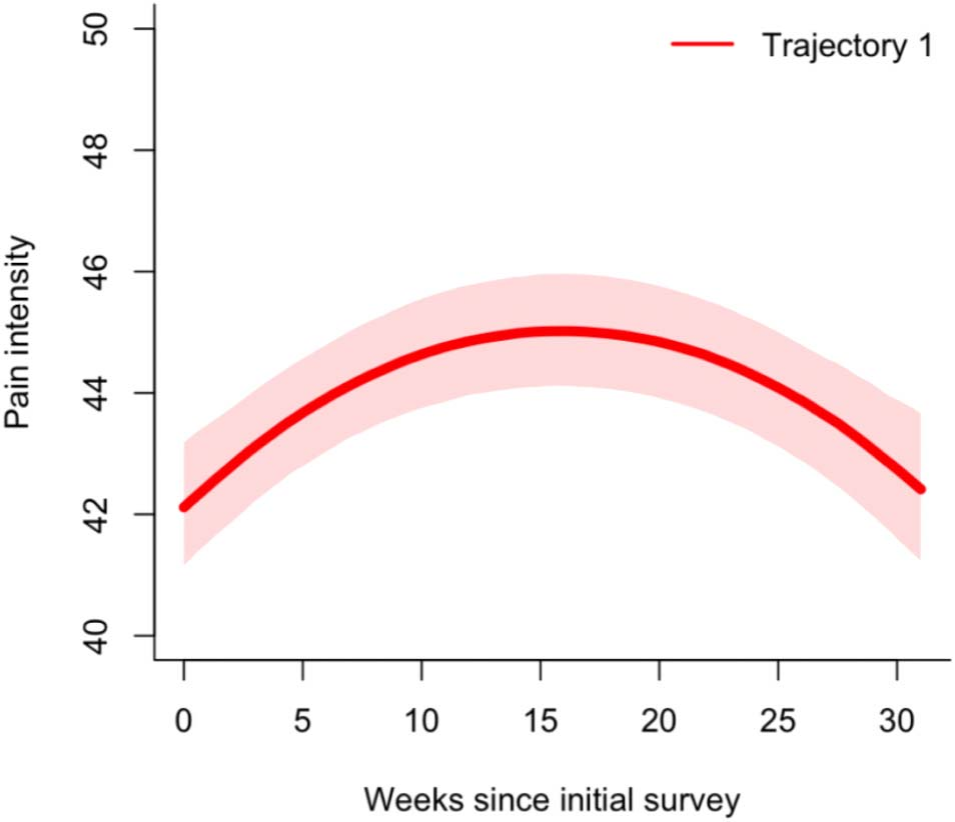
Pain intensity trajectory. Shaded areas represent 95% confidence intervals. Clinical cut-offs/scores above population averages: Pain intensity t-scores above 60.

The final model consisted of a quadratic term, with a single trajectory group and random intercept, and 5 equidistant knots (Fig. 1). The single trajectory group remained stable and within population averages of pain intensity, despite an increased proportion of reported pain intensity above population averages by 11% in the second trimester and 16% in the third trimester as compared to the first trimester. As a single trajectory best modeled pain intensity in this population, no analyses of posterior probability and calculation of baseline factors, lasso, or imputation were performed.

#### 3.3.2. Pain catastrophizing modeling

After fitting the trajectory models, the summed AIC and BIC indicated that a 2-group model best fit the data, with a near even distribution of participants between the 2 groups (Supplemental Table S2, http://links.lww.com/PR9/A219).

**Figure 2.**
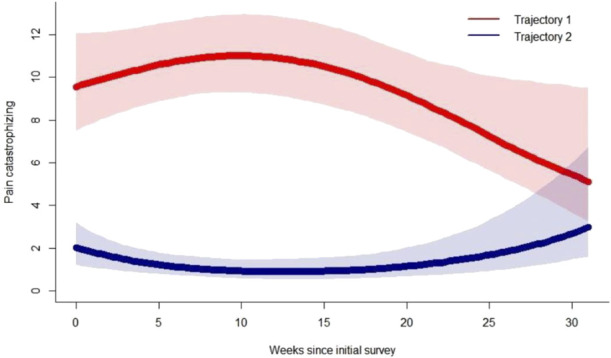
Pain catastrophizing trajectories. Trajectory 1 (red) represents the *moderate pain catastrophizing group*. Trajectory 2 (blue) represents the *no pain catastrophizing group.* Shaded areas represent the 95% confidence intervals. Clinical cut-offs/scores above population averages: Pain Catastrophizing (PCS) total score above 30.

The final model consisted of a quadratic term, with 2 trajectory groups, random intercept, and slope, and 3 quantile knots (Fig. 2). To see plots of individual specific data points, please refer to Supplemental Figure S1, http://links.lww.com/PR9/A219. The *moderate pain catastrophizing* group (N = 69, 49%) entered the study with pain catastrophizing below the clinically significant range (PCS total score <30), which remained stable before declining postpartum. The *no pain catastrophizing* group (N = 71, 51%) entered the study with a median PCS score of 0. The convergence of 95% CIs is indicative of decreasing pain catastrophizing and a reduction in differences between trajectory groups postpartum. The average posterior probabilities for the individual groups were 0.93 for the *moderate pain catastrophizing group* and 0.91 for the *no pain catastrophizing group*, which exceed the recommended acceptable posterior probability of 0.70.

#### 3.3.3. Pain catastrophizing trajectory characteristics

Baseline characteristics of patients by pain catastrophizing trajectory are provided in Table 3. Income and baseline measures of pain catastrophizing, pain interference, pain intensity, anxiety, and depression were significantly different between trajectory groups.

**Table 3:** Pain catastrophizing trajectory characteristics.

	Trajectory 1 *Moderate pain catastrophizing* (N = 69, 49%)	Trajectory 2 *No pain catastrophizing* (N = 71, 51%)	*P*
Age (y)	31.0 [28.0–33.5]	31.0 [29.0–35.0]	0.407*
Body mass index (kg/m^2^)	23.2 [20.1–27.3]	23.5 [21.4–26.5]	0.656*
Ethnicity			0.087†
Caucasian	39 (57)	50 (70)	
Other	30 (43)	21 (30)	
Income	11.0 [8.0–16.0]	14.0 [9.0–21.0]	0.041*‡
Education			0.195†
High School or less	7 (10)	4 (6)	
Postsecondary	44 (64)	39 (55)	
Master's/Doctorate	18 (26)	38 (39)	
Number of children	0.0 [0.0–1.0]	0.0 [0.0–1.0]	0.291*
Baseline pain catastrophizing	6.0 [3.0–14.0]	0.0 [0.0–4.0]	<0.001*‡
Baseline postnatal depression score	7.0 [5.0–11.0]	4.0 [2.0–6.0]	<0.001*‡
Baseline pain interference score	55.6 [52.5–60.9]	49.6 [41.6–55.6]	<0.001*‡
Baseline pain intensity score	43.5 [40.2–46.3]	40.2 [40.2–43.5]	<0.001*‡
Baseline anxiety score	3.0 [1.0–5.80]	1.5 [0.0–3.0]	<0.001*‡
Baseline Insomnia Severity Index	9.0 [4.0–13.0]	6.0 [2.0–8.0]	0.001*‡

Pain catastrophizing at baseline and across all time points was missing for 2 participants; thus, N = 140 for pain catastrophizing trajectory analysis.

* Continuous data presented as median [interquartile range] per finding of nonnormality (Shapiro–Wilk test, *P* < 0.05), based on Mann–Whitney *U*.

† Categorical data presented as frequency (percentage), based on χ^2^ tests.

‡ Statistical significance (*P* < 0.05).

N, number of participants.

#### 3.3.4. Associations of pain catastrophizing trajectory membership

Significant individual associations of trajectory membership included symptoms of anxiety and depression, insomnia, pain interference, and pain intensity (Table 4). Relative to the *no pain catastrophizing* group, higher anxiety and depressive symptoms at first assessment were associated with increased odds of belonging to the *moderate pain catastrophizing* group (OR = 1.44 [1.21–1.71], OR = 1.25 [1.13–1.39], respectively, *P* < 0.001). Having insomnia was also related to higher odds of belonging to the *moderate pain catastrophizing* group (OR = 1.12 [1.04–1.20], *P* = 0.003). Finally, relative to the *no pain catastrophizing* group, higher reports of pain interference and pain intensity at first assessment increased the odds of belonging to the *moderate pain catastrophizing* group (OR = 1.12 [1.06–1.17], OR = 1.15 [1.07–1.24], respectively, *P* < 0.001). Table 5 provides the complete odds ratios and *P* values.

**Table 4:** Associations with pain catastrophizing trajectory membership.

	Trajectory 1 (in reference trajectory 2) Odds ratio (95% confidence interval)	*P*
Age (y)	0.96 (0.89–1.04)	0.296
Body mass index (kg/m^2^)	1.00 (0.94–1.06)	0.919
Ethnicity		
Caucasian	Reference	Reference
Other	1.83 (0.91–3.68)	0.089
Income	0.95 (0.90–1.00)	0.029*
Education		
High School or less	1.55 (0.42–5.70)	0.509
Postsecondary	Reference	Reference
Master's/Doctorate	0.57 (0.27–1.19)	0.132
No. of children	0.74 (0.45–1.22)	0.240
Baseline pain catastrophizing score	1.27 (1.16–1.41)	<0.001*
Baseline postnatal depression score	1.25 (1.13–1.39)	<0.001*
Baseline pain interference score	1.12 (1.06–1.17)	<0.001*
Baseline pain intensity score	1.15 (1.07–1.24)	<0.001*
Baseline anxiety score	1.44 (1.21–1.71)	<0.001*
Baseline Insomnia Severity Index	1.12 (1.04–1.20)	0.003*

Trajectory 1 = moderate pain catastrophizing group.

Trajectory 2 = no pain catastrophizing group.

* Statistical significance (*P* < 0.05).

**Table 5:** Associations between baseline characteristics and pain catastrophizing trajectory group.

	*Complete case (N = 129)		Imputed case (N = 142)	
	Estimate (95% CI)	*P*	Estimate (95% CI)	*P*
Intercept	7.45 × 10^−3^ (5.84 × 10^−4^ to 6.63 × 10^−2^)	<0.001†	1.01 × 10^−2^ (1.20 × 10^−3^ to 8.57 × 10^−2^)	<0.001†
Ethnicity	2.01 (0.72–5.88)	0.188	1.63 (0.62–4.30)	0.319
Number of children	0.71 (0.33–1.42)	0.349	0.79 (0.42–1.49)	0.466
Baseline pain intensity	1.70 (1.21–2.48)	<0.001†	1.61 (1.16–2.23)	0.005†
Baseline anxiety	1.35 (1.10–1.74)	<0.001†	1.36 (1.10–1.68)	0.005†
Baseline pain catastrophizing	1.20 (1.08–1.36)	<0.001†	1.22 (1.09–1.36)	<0.001†
Model summary	AUC (95% CI): 0.85 (0.77–0.91)		AUC (95% CI): 0.86 (0.78–0.91)	

Independent factors to adjust for associations between trajectory groups were selected using least absolute shrinkage and selection operator (LASSO) regression. Trajectory group 2 (the no pain catastrophizing group) was the reference group. Non-zero coefficients as found by LASSO regression were subsequently transferred to a multivariable logistic regression.

* Complete cases represent those with available data for all listed model variables.

† Statistical significance (*P* < 0.05).

AUC, area under the curve; CI, confidence interval; N, number of participants.

#### 3.3.5. Pain catastrophizing lasso and imputation

Non-zero coefficients of pain catastrophizing trajectory groups included ethnicity, number of children, baseline pain intensity, anxiety, and pain catastrophizing. To assess whether trajectory groups could accurately discriminate women with no and moderate pain catastrophizing, model sensitivity and specificity were calculated as 74.6 and 85.5, respectively. The area under the receiver operating characteristic (ROC) curve was 0.848 (CI: 0.772–0.910). Results of multivariable logistic regression after imputation are presented in Table 5.

#### 3.3.6. Pain interference modeling

After fitting the trajectory models, the summed AIC and BIC indicated that a 3-group model best fit the data (see Supplemental Table S3, http://links.lww.com/PR9/A219).

**Figure 3.**
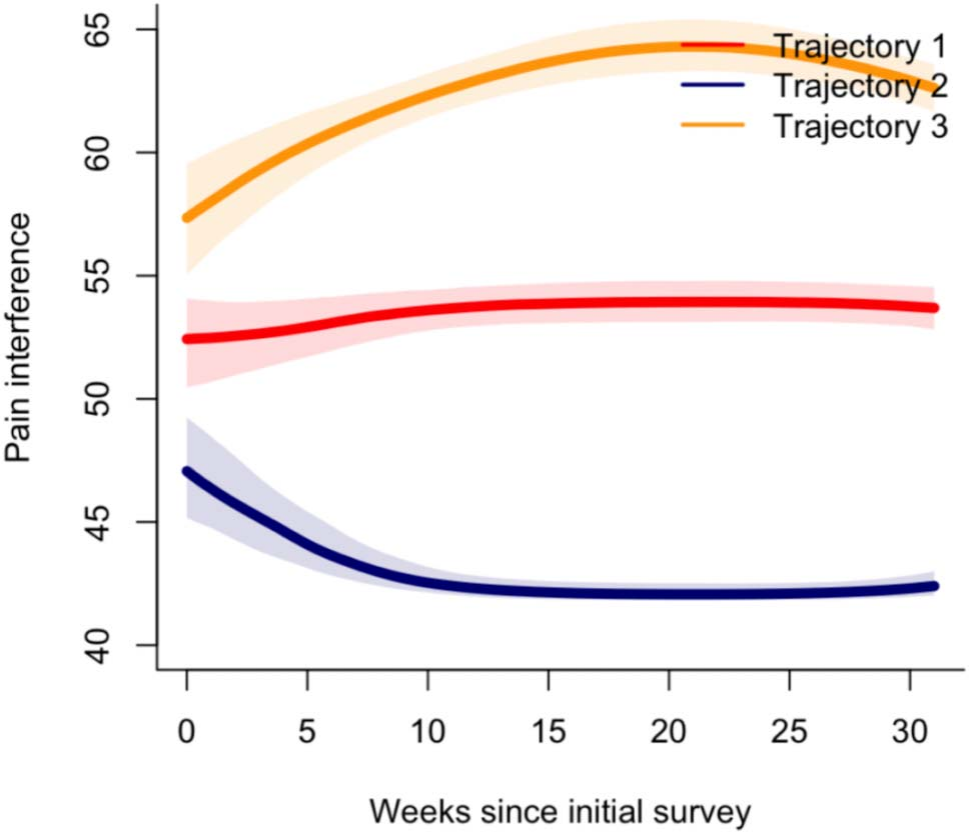
Pain interference trajectories. Trajectory 1 (red) represents the *moderate pain interference group*. Trajectory 2 (blue) represents the *no pain interference group.* Trajectory 3 (yellow) represents the *high pain interference group.* Shaded areas represent the 95% confidence intervals. Clinical cut-offs/scores above population averages: Pain interference (PROMIS) t-scores above 60.

The final model consisted of a quadratic term, with 3 trajectory groups, random intercept, and slope, and 5 equidistant knots (Fig. 3). To see plots of individual specific data points, please refer to Supplemental Figure S2, http://links.lww.com/PR9/A219. The *moderate pain interference* group (N = 68, 48%) consistently reported pain interference within population averages. The *no pain interference* group (N = 31, 22%) entered the study with scores below the population average, which were consistently maintained into the postpartum assessment. The *high pain interference* group (N = 43, 30%) entered the study with scores within population average, which then increased above population averages (Patient-Reported Outcomes Measurement Information System Pain Interference score >60) in the second and third trimester, and were consistently maintained into the postpartum assessment. The average posterior probabilities for the individual groups were 0.93 for the *moderate pain interference group,* 0.95 for the *no pain interference group*, and 0.93 for the *high pain interference group*, which exceed the recommended acceptable posterior probability of 0.70.

#### 3.3.7. Pain interference trajectory characteristics

Baseline characteristics of participants by pain interference trajectory are given in Table 6. Ethnicity, baseline pain catastrophizing, anxiety, pain intensity, depression, pain interference, and insomnia differed between the trajectory groups.

**Table 6:** Pain interference trajectory characteristics.

	Trajectory 1 *Moderate pain interference group* (N = 68, 48%)	Trajectory 2 *No pain interference group* (N = 31, 22%)	Trajectory 3 *High pain interference group* (N = 43, 30%)	*P*
Age (y)	30.0 [28.0–35.0]	32.0 [29.5–35.0]	31.0 [28.5–34.0]	0.411*
Body mass index (kg/m^2^)	23.4 [21.9–26.8]	23.7 [21.4–26.5]	22.7 [19.8–26.3]	0.328*
Ethnicity				0.021†‡§‖
Caucasian	51 (75)	17 (55)	22 (51)	
Other	17 (25)	14 (45)	21 (49)	
Income	12.0 [8.0–20.0]	14.0 [10.0–20.5]	11.0 [8.0–14.0]	0.293*
Education				0.807‡
High school or less	6 (9)	1 (3)	5 (12)	
Postsecondary	42 (61)	20 (65)	21 (49)	
Master's/Doctorate	20 (29)	10 (32)	17 (39)	
No. of children	0.0 [0.0–1.0]	1.0 [0.0–1.0]	0.0 [0.0–1.0]	0.123*
Baseline pain catastrophizing	3.0 [0.0–8.0]	0.0 [0.0–4.0]	5.0 [2.0–12.0]	<0.001†*§#
Baseline postnatal depression score	6.0 [3.0–8.0]	3.0 [1.0–5.0]	7.0 [5.0–11.0]	<0.001†*§‖#
Baseline pain interference score	53.9 [49.6–55.6]	41.6 [41.6–54.8]	58.8 [53.9–62.5]	<0.001†*§‖#
Baseline pain intensity score	43.5 [40.2–46.3]	40.2 [30.7–43.5]	43.5 [40.2–47.1]	0.002†*#
Baseline anxiety score	2.0 [1.0–4.0]	1.0 [0.0–3.0]	3.0 [1.0–6.0]	0.005†*§#
Baseline Insomnia Severity Index	7.0 [4.0–9.5]	4.0 [1.0–6.0]	10.0 [4.50–14.0]	<0.001†*§‖#

* Continuous data presented as median [interquartile range] per finding of nonnormality (Shapiro–Wilk test, *P* < 0.05), based on Kruskal–Wallis.

† Statistical significance (*P* < 0.05).

‡ Categorical data presented as frequency (percentage), based on χ^2^ tests.

§ Significantly different between trajectory 1 and 2 by the Mann–Whitney *U* Test (*P* < 0.05).

‖ Significantly different between trajectory 1 and 3 by the Mann–Whitney *U* Test (*P* < 0.05).

# Significantly different between trajectory 2 and 3 by the Mann–Whitney *U* Test (*P* < 0.05).

N, number of participants.

#### 3.3.8. Associations of pain interference trajectory membership

Significant associations with the *no pain interference* trajectory membership include ethnicity, baseline pain catastrophizing, depression, pain interference, pain intensity, anxiety, and insomnia. The odds of following the *no pain interference* trajectory group were decreased for participants with ethnicities other than Caucasian (OR = 0.405 [0.165–0.99], *P* = 0.048) and were increased for women with multiple children (OR = 1.86 [1.02–3.38], *P* = 0.044). The odds of following the *no pain interference* trajectory group additionally decreased for every unit increase in baseline pain catastrophizing (OR = 0.875 [0.783–0.977], *P* = 0.018), depressive symptoms (OR = 0.752 [0.635–0.891], *P* < 0.001), pain interference (OR = 0.878 [0.820–0.939], *P* < 0.001), pain intensity (OR = 0.911 [0.839–0.989], *P* = 0.026), anxiety (OR = 0.763 [0.602–0.966], *P* < 0.025), and insomnia (OR = 0.861 [0.767–0.967], *P* < 0.011). Relative to the *moderate pain interference* group, higher pain catastrophizing (OR = 1.06 [1.003–1.11], *P* = 0.037), depressive symptoms (OR = 1.12 [1.03–1.22], *P* = 0.010), pain interference (OR = 1.10 [1.04–1.17], *P* = 0.002), pain intensity (OR = 1.13 [1.03–1.23], *P* = 0.010), and insomnia (OR = 1.09 [1.01–1.18], *P* = 0.034) at first assessment were associated with increased odds of belonging to the *high pain interference* group. Refer to Table 7 for complete odds ratios and *P* values.

**Table 7:** Associations with pain interference trajectory membership.

	Trajectory 2 (reference Trajectory 1) Odds ratio (95% confidence interval)	*P*	Trajectory 3 (reference Trajectory 1) Odds ratio (95% confidence interval)	*P*
Age (y)	1.04 (0.99–1.19)	0.099	1.01 (0.93–1.11)	0.769
Body mass index (kg/m^2^)	1.01 (0.93–1.09)	0.830	0.96 (0.88–1.04)	0.278
Ethnicity				
Caucasian	Reference	Reference	Reference	Reference
Other	0.41 (0.17–0.99)	0.048*	0.35 (0.16–0.79)	0.011*
Income	1.01 (0.95–1.07)	0.747	0.96 (0.91–1.01)	0.141
Education				
High school or less	1.38 (0.48–3.92)	0.551	1.15 (0.44–3.02)	0.771
Postsecondary	Reference	Reference	Reference	Reference
Master's/Doctorate	1.00 (0.36–2.75)	1.000	0.68 (0.27–1.68)	0.402
No. of children	1.86 (1.02–3.38)	0.044*	1.07 (0.59–1.94)	0.837
Baseline pain catastrophizing score	0.88 (0.78–0.98)	0.018*	1.06 (1.00–1.11)	0.037*
Baseline postnatal depression score	0.75 (0.64–0.89)	<0.001*	1.12 (1.03–1.22)	0.010*
Baseline pain interference score	0.88 (0.82–0.94)	<0.001*	1.10 (1.04–1.17)	0.002*
Baseline pain intensity score	0.91 (0.84–0.99)	0.026*	1.13 (1.03–1.23)	0.010*
Baseline anxiety score	0.76 (0.60–0.97)	0.025*	1.07 (0.95–1.19)	0.277
Baseline Insomnia Severity Index	0.86 (0.77–0.97)	0.011*	1.09 (1.01–1.18)	0.034*

Trajectory 1 = moderate pain interference group.

Trajectory 2 = no pain interference group.

Trajectory 3 = high pain interference group.

* Statistical significance (*P* < 0.05).

#### 3.3.9. Pain interference lasso and imputation

Non-zero coefficients of pain interference trajectory groups included baseline depression, pain interference, and pain intensity. The area under the ROC curve for trajectory 2 was 0.799 (0.705–0.881), and the area under the ROC curve for trajectory 3 was 0.760 (0.658–0.834). Results of multinomial regression after imputation are presented in Table 8.

**Table 8:** Associations between baseline characteristics and pain interference trajectory group.

Trajectory 2					Trajectory 3			
	*Complete case (N = 129)		Imputed case (N = 142)		*Complete case (N = 129)		Imputed case (N = 142)	
	Estimate (95% CI)	*P*	Estimate (95% CI)	*P*	Estimate (95% CI)	*P*	Estimate (95% CI)	*P*
Intercept	1.41 × 10^2^ (1.68–1.19 × 10^4^)	0.029†	3.12 ×10^2^ (4.54–2.14 × 10^4^)	0.008†	2.03 ×10^−4^ (1.06 × 10^−6^ to 3.88 × 10^−2^)	0.002†	6.58 ×10^−4^ (7.23 × 10^−6^ to 5.98 × 10^−2^)	0.002†
Baseline pain intensity	0.96 (0.87–1.06)	0.440	0.98 (0.89–1.08)	0.686	1.10 (0.98–1.24)	0.097	1.05 (0.96–1.17)	0.247
Baseline depression	0.81 (0.68–0.97)	0.024†	0.78 (0.66–0.93)	0.007†	1.05 (0.95–1.16)	0.328	1.08 (0.99–1.18)	0.094
Baseline pain interference	0.94 (0.87–1.01)	0.097	0.91 (0.84–0.98)	0.015†	1.06 (0.99–1.15)	0.115	1.07 (1.00–1.15)	0.045
Model summary	AUC (95% CI): 0.80 (0.71–0.88)		AUC (95% CI): 0.84 (0.74–0.90)		AUC (95% CI): 0.76 (0.66–0.83)		AUC (95% CI): 0.78 (0.68–0.85)	

Trajectory 1 = moderate pain interference group.

Trajectory 2 = no pain interference group.

Trajectory 3 = high pain interference group.

Complete case: AUC of trajectory 1 compared with trajectory 2 and 3: 0.61 (0.51–0.71).

Imputed case: AUC of trajectory 1 compared with trajectory 2 and 3: 0.49 (0.38–0.60).

Independent predictors to adjust for associations between trajectory groups were selected using least absolute shrinkage and selection operator (LASSO) regression. Trajectory group 1 was the reference group. Non-zero coefficients as found by LASSO regression were subsequently transferred to a multivariable multinomial logistic regression model.

* Complete cases represent those with available data for all listed model variables.

† Statistical significance (*P* < 0.05).

AUC, area under the curve; CI, confidence interval; N, number of participants.

## 4. Discussion

These exploratory analyses identified distinct trajectories of pain catastrophizing and pain interference from early pregnancy to postpartum. Two trajectories of pain catastrophizing emerged, and 3 trajectories of pain interference were identified. Overall, reports of pain intensity, pain catastrophizing, and pain interference remained subclinical for a large proportion of participants. This suggests that the propensity for pain to interfere with everyday functioning is relatively low in this population. The subset of individuals reporting higher pain symptomology at initial assessment were more likely to report an increase across all pain measures during the third trimester.

Pain intensity is a highly used pain metric in clinical care and research settings.^19,27^ However, there were no discernible heterogeneous trajectories across pregnancy and into the postpartum, indicating that the pain intensity experience of women was relatively homogeneous across our sample. Clinically significant reports of pain intensity were greatest in the third trimester, with 20.6% of our sample scoring above clinical cutoffs. However, assigning objective value to the subjective pain experience is challenging. This is particularly true when trying to assign a clinical value to single-item pain scales.^26^ Single-item pain measures are inadequate in describing pain experience, which may explain the homogeneity in reported pain intensity across our sample. Multiple facets of the pain experience should be used to evaluate pain symptomology more holistically.

Pain catastrophizing represents the extent that individuals ruminate, magnify, and feel helpless in response to pain. In pregnant populations, pain catastrophizing has been associated with fear of birth, increased incidence of depression and anxiety, poorer physical ability, and poorer postpartum recovery.^16,20,37,47^ Although pain catastrophizing symptoms remained low for a substantial proportion of women throughout pregnancy, women with greater pain interference, pain intensity, anxiety, depression, and insomnia at baseline were more likely to belong to the *moderate pain catastrophizing* trajectory group. Therefore, the *moderate pain catastrophizing group* may be at increased risk for concurrent internalizing mental health conditions and pain symptomology. Individuals in the *moderate pain catastrophizing group* may benefit from symptom monitoring and anticipatory interventions to manage pain catastrophizing symptoms. Identification of pregnant women at risk for pain catastrophizing and the timely introduction of pain interventions may improve postpartum recovery outcomes.

Pain interference refers to the extent that pain disrupts daily routines, social, and physical activities.^28^ Reports of pain interference in pregnancy have been associated with poorer work functioning and difficulty in both physical and daily activities.^6,14^ Exploration of pain interference in pregnant samples has been limited; however, previous literature has indicated that pregnant women with higher reports of pain interference are more likely to report greater pain intensity and pain catastrophizing.^10^ In the present study, women experiencing higher pain catastrophizing, pain intensity, depressive symptoms, anxiety symptoms, and insomnia were more likely to belong to the *moderate* or *high pain interference* trajectory group, as compared to the *no pain interference* trajectory group. Close to half of all participants belonged to the *moderate pain interference group*. Moreover, 30 percent of women belonged to the *high pain interference group*, reporting clinically significant pain interference in the second and third trimesters. Women in both the *moderate* and *high pain interference groups* may be at risk for disruptions to activities of daily living and could perhaps benefit from symptom monitoring and anticipatory treatment to reduce negative consequences of pain interference. Early identification of pregnant women at risk for developing clinically significant pain interference could allow for the introduction of appropriate pain coping strategies, which may reduce the burden of pain-related disability and improve their quality of life.

This study demonstrated heterogeneous groups of pain catastrophizing and pain interference, and associations with internalizing mental health indices during a critical time of infant development, which may have implications for both mothers and their children. Increased pain incidence during pregnancy has been associated with a reduction in quality of life, greater report of internalizing mental health conditions, and adverse labor and delivery outcomes.^7,18,48^ Clinical findings of both prenatal and postnatal stress and maternal somatic complaints have been associated with increased child somatization at 18 months.^54^ Similarly, increased maternal stress hormone levels during pregnancy have been associated with worse behavioral stress recovery and larger cortisol responses to a painful procedure in infants aged 2 years.^15^ Therefore, maternal prenatal stress may increase the risk of development of comorbid somatization and internalizing problems in infants.

There are several limitations to this exploratory work. Although the sample size reached the minimum threshold for GMM, and is comparable to other studies using GMM, we recognize that this sample size is on the lower end of what is acceptable for this analysis.^39,44^ Although this study generated meaningful results to inform future work, conclusions drawn from our findings must be interpreted with caution. A larger sample size may improve discriminatory capacity of our models to detect differences in pain experience.

This work was a secondary analysis of prenatal and postpartum data^48^ and would have benefited from additional measures to characterize pain. For example, pain history is an important consideration, given that a previous history of chronic pain is a known risk factor for the development of pain during pregnancy.^34^ Recording pain locations is important to discern whether a new pain problem has emerged or whether the reported pain is persistent. There is limited work exploring changes in the pain type during pregnancy vs the postpartum period, which is an important direction for future research. We also do not have information on pain medication use or pain treatment during the prenatal and postpartum period. The use of several commonly prescribed pain medications during pregnancy has been proven to be safe and effective^3^ and could potentially alter pain experience. Therefore, pain medication use during pregnancy is an important consideration for future work characterizing pain during pregnancy. In addition, excessive weight gain during pregnancy has been linked to enhanced stress on the musculoskeletal system^32^ and has been associated with persistent pain in the postpartum.^52^ Therefore, weight gain during pregnancy should be considered.

The participants in our study were predominantly White and of higher socioeconomic status (SES). Lower and moderate SES groups are at higher risk for chronic pain development, and pregnant lower SES women specifically are at higher risk of adverse pregnancy outcomes.^30,40^ Replication of the present study in more diverse samples is an important future research consideration. Furthermore, as all women in this study were considered low-risk pregnancies, future work in high-risk pregnancies and pregnant women with comorbidities or clinically diagnosed internalizing mental health conditions should be studied to ensure the generalizability of the findings from this work.

In summary, we demonstrated heterogeneity in pain catastrophizing and pain interference symptomology from pregnancy into the postpartum. Although a small subset of women experienced clinically significant pain catastrophizing, a larger identifiable group of women experienced clinically significant pain interference. These findings highlight the prevalence of pain throughout pregnancy and reinforce the need for regular pain assessment and management, as a part of pregnancy and postnatal care.

## Disclosures

The authors have no conflict of interest to declare.

## Supplementary Material

## Supplemental digital content

Supplemental digital content associated with this article can be found online at http://links.lww.com/PR9/A219.
