# Supplementary material for: Trajectories of pain intensity, pain catastrophizing, and pain interference in the perinatal and postpartum period
Source: Pain Rep. 2024 Jan 7;9(2):e1137. doi: 10.1097/PR9.0000000000001137 (PMC10852363; doi:10.1097/PR9.0000000000001137)
Supplement: Supplementary file 1 [file painreports-9-e1137-s001.pdf]

Appendix A: Full Description of Statistical Analyses

Trajectory Classes and Model Selection. Trajectory analyses utilized latent class mixed modelling (LCMM 1.7.8 package in R) [6]. LCMM assumes a heterogenous population with recognizable trajectories over time. LCMM allows for analyses with missing data points [4]. Fifty models were individually developed for pain intensity, pain catastrophizing and pain interference. Model development included: 1) the consideration of the number of distinct trajectory groups (1 to 5), 2) the investigation of linear and quadratic spline models (2 to 5 knots), and 3) the incorporation of a quadratic temporal term. Linear and quadratic temporal terms, as well as the participant were considered as random effects [7]. Growth mixture modelling allows for improved flexibility. One such modelling feature is the inclusion of individual intercepts and slopes as random effects. As such we included linear and non-linear time terms as random effects (specifically to model individual slopes) to assess their impact on model fit. Model fit was evaluated using both the Akaike information criterion (AIC) and the Bayesian information criteria (BIC). The AIC indicates the statistical parameter at which the model complexity outweighs the model fit. This is similar to the BIC, which evaluates models with a more stringent threshold towards penalization of model complexity. Model fit improvement amongst developed models was indicated by a difference in 10 for both AIC and BIC diagnostics [1]. Identification of the best model fit was determined by the lowest sum of the AIC and the BIC, with a minimum class membership of 5%. Following identification of best model fit, the posterior probabilities of membership for each trajectory class were determined.

Lasso and Imputation Lasso and Imputation. Multivariable complete case models to predict baseline characteristics associated with trajectory membership were developed using least absolute shrinkage and selection operator (lasso) regression. Multivariable model development to predict trajectory membership was developed using lasso. Adaptive lasso was utilized for outcomes with two trajectories, whereas multinomial lasso was used for outcomes with greater than two trajectories (glmnet 4.1-8 package in R) [3]. To ensure appropriate variable selection during the lasso procedures ethnicity and education were recoded into binary variables. A correlation plot was first generated to assess continuous variables for high correlation (>0.7). This was followed by an initial ridge regression using 10-fold cross validation – a form of

penalized regression to calculate corresponding penalty factors for each included variable. Following ridge regression, a lasso regression with 10-fold cross validation was completed. The calculated penalty factors from ridge regression were incorporated into the lasso procedure. Unlike ridge regression, lasso is a form of penalization regression that can shrink coefficients to zero allowing it to function as a variable selection tool. The degree of penalization was fine-tuned using the regularization parameter lambda [8]. When lambda is set to zero, the penalty term has no effect, and the coefficient is not reduced. However, as lambda increases, the degree of penalization increases, and regression coefficients approach zero. The largest value of lambda utilized was within 1-standard error (SE) of the cross-validated error for the minimum lambda. The use of lambda within 1-SE of the minimum typically results in more parsimonious model, which generates a model with higher bias, but lower variance. Non-zero regression coefficients resultant from lasso regression were transferred to an unrestricted logistic or multinomial logistic regression for final model development. Area under the receiver operating characteristic (ROC) curve of the models were subsequently presented to assess model performance.

Missing data imputation was used to complete a sensitivity analysis of the complete case logistic and multinomial multivariable models (MICE 3.16.0 package in R) [2]. Little's test ($p<0.001$) showed that data was not missing completely at random, however this test does not discern whether data is missing at random or not missing at random. Although MICE imputation assumes that data is missing at random, it has been demonstrated that even when data is not missing at random, multiple imputation produces more favourable results than listwise deletion, which is more likely to lead to bias [5]. Using demographic and baseline variables and assuming data was missing at random, 8 models were imputed to match the variable with the highest degree of missingness using multiple imputed chained equations (MICE) with predictive mean matching and logistic regression. Imputed models were subsequently pooled, and logistic and multinomial models constructed using equivalent variables to those selected via lasso on our complete case data. Both complete case and imputed case models were presented using odds ratios, 95% CI and p-values.

Supplemental Figures

Figure S1. Individual specific data points for Pain Catastrophizing trajectories. Trajectory 1 (red) represents the *moderate pain catastrophizing group,* Trajectory 2 (blue) represents the *no pain catastrophizing group.* Jitter has been added to this figure to improve visual presentation.

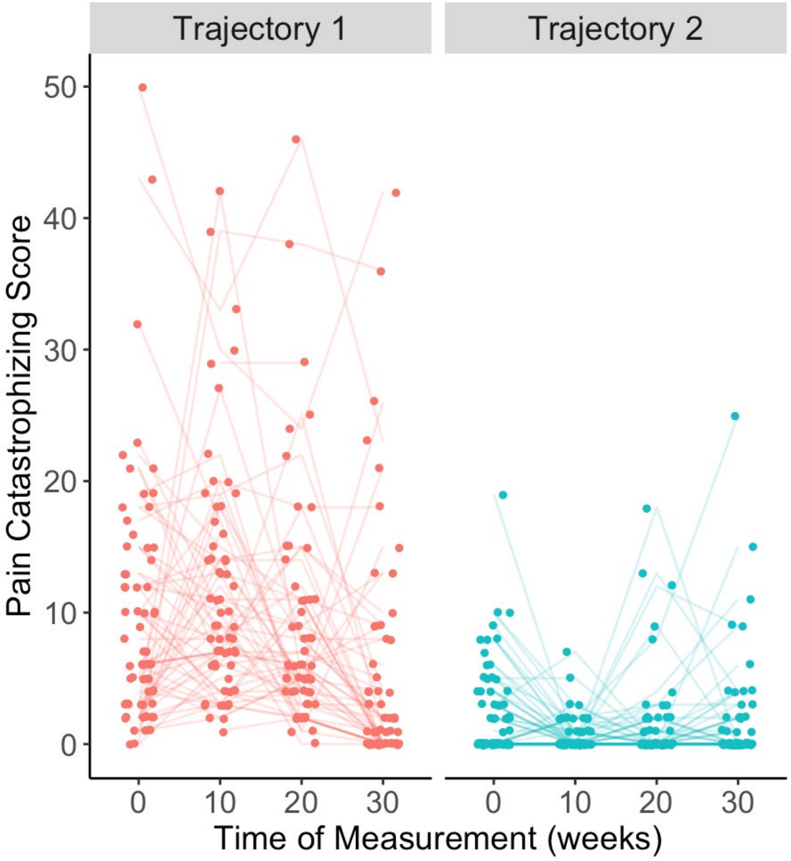

Figure S2. Individual specific data points for Pain Interference trajectories. Trajectory 1 (red) represents the *moderate pain interference group,* Trajectory 2 (green) represents the *no pain interference group.* Trajectory 3 (blue) represents the *high pain interference group.* Jitter has been added to this figure to improve visual presentation.

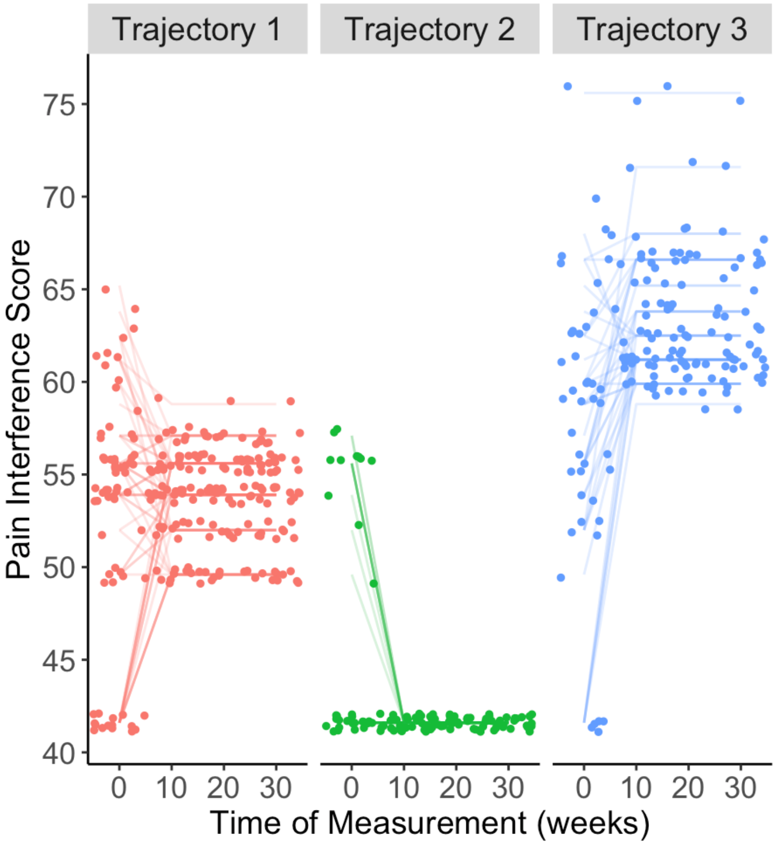

Supplemental Tables

Table S1. Pain Intensity model fit diagnostics (requirement of ≥ 5% trajectory membership)

**Number of Trajectories**	[a]**Linear**		[a]**Quadratic**		**Percentage per Trajectory**	
	[b]AIC	BIC	AIC	BIC	Linear	Quadratic
Without random effects						
1	3082	3105	3069	3095	100	100
2	3025	3058	3006	3045	61 | 39	35 | 65
3	3005	3046	2892	3032	14 | 70 | 16	17 | 67 | 16
4	3008	3058	2984	3046	3 | 64 | 18 | 15	12 | 58 | 12 | 18
5	3015	3073	2985	3058	3 | 16 | 1 | 64 | 16	9 | 55 | 15 | 6 | 15
With random intercept						
**1**	3003	3029	**2978**	**3007**	100	**100**
2	3005	3040	2977	3018	39 | 61	16 | 84
3	3006	3050	2978	3031	71 | 13 | 16	11 | 70 | 19
4	3010	3063	2983	3048	3 | 18 | 64 | 15	5 | 10 | 13 | 72
5	3016	3078	2986	3063	23 | 3 | 19 | 38 | 17	12 | 56 | 4 | 15 | 13

AIC, Akaike Information Criterion; BIC, Bayesian Information Criterion
[a]Time (weeks) since initial survey considered as either a linear (time) or linear + quadratic term (time + $time^2$)
[b]Using sum of AIC and BIC to determine best model

Table S2 Pain Catastrophizing model fit diagnostics (requirement of ≥ 5% trajectory membership)

**Number of Trajectories**	[a]**Linear**		[a]**Quadratic**		**Percentage per Trajectory**	
	[b]AIC	BIC	AIC	BIC	Linear	Quadratic
Without random effects						
1	2605	2623	2605	2626	100	100
2	2487	2514	2482	2515	49 | 51	50 | 50
3	2451	2486	2448	2492	4 | 52 | 44	4 | 51 | 45
4	2448	2492	2450	2506	3 | 20 | 39 | 38	4 | 47 | 18 | 31
5	2450	2503	2454	2522	3 | 24 | 34 | 36 | 3	21 | 30 | 15 | 8 | 26
With random intercept and slope						
1	2466	2492	2466	2504	100	100
**2**	2465	2501	**2456**	**2506**	59 | 41	**49 | 51**
3	2466	2511	2463	2525	6 | 94 | 0	0 | 19 | 81
4	2458	2511	2456	2529	56 | 4 | 40 |0	13 | 37 | 15 | 35
5	2455	2517	2450	2536	3 | 37 | 21 | 3 | 36	27 | 22 | 1 | 22 | 27

AIC, Akaike Information Criterion; BIC, Bayesian Information Criterion
[a]Time (weeks) since initial survey considered as either a linear (time) or linear + quadratic term (time + $time^2$)
[b]Using sum of AIC and BIC to determine best model

Table S3. Pain Interference model fit diagnostics (requirement of ≥ 5% trajectory membership)

**Number of Trajectories**	[a]**Linear**		[a]**Quadratic**		**Percentage per Trajectory**	
	[b]AIC	BIC	AIC	BIC	Linear	Quadratic
Without random effects						
1	3477	3501	3478	3505	100	100
2	3233	3265	3224	3263	74 | 26	31 | 69
3	3090	3132	3071	3121	29 | 49 | 22	29 | 49 | 22
4	3061	3111	2943	3005	28 | 13 | 37 | 22	27 | 37 | 16 | 20
5	3026	3085	2708	2782	37 | 27 | 14 | 8 | 14	27 | 37 | 16 | 8 | 12
With random intercept and slope						
1	3026	3058	2638	2682	100	100
**2**	3029	3070	2643	2699	87 | 13	79 | 21
3	3030	3080	**2628**	**2696**	11 | 73 | 16	**48 | 22 | 30**
4	3025	3085	2621	2701	63 | 8 | 14 | 15	22 | 15 | 59 | 4
5	3006	3074	2635	2727	26 | 8 | 38 | 14 | 14	22 | 9 | 35 | 30 | 4

AIC, Akaike Information Criterion; BIC, Bayesian Information Criterion

[a]Time (weeks) since initial survey considered as either a linear (time) or linear + quadratic term (time + $time^2$)

[b]Using sum of AIC and BIC to determine best model

References

[1] Burnham KP, Anderson DR. Multimodel Inference: Understanding AIC and BIC in Model Selection. Sociol Methods Res 2004;33:261–304.

[2] Buuren S, Groothuis-Oudshoorn C. MICE: Multivariate Imputation by Chained Equations in R. J Stat Softw 2011;45.

[3] Friedman J, Hastie T, Tibshirani R, Narasimhan B, Tay K, Simon N, Qian J. Package 'glmnet.' CRAN R Repositary 2021;595.

[4] Gardner RC, Cheng J, Ferguson AR, Boylan R, Boscardin J, Zafonte RD, Manley GT, Citicoline Brain Injury Treatment Trial Investigators. Divergent Six Month Functional Recovery Trajectories and Predictors after Traumatic Brain Injury: Novel Insights from the Citicoline Brain Injury Treatment Trial Study. J Neurotrauma 2019;36:2521–2532.

[5] van Ginkel JR, Linting M, Rippe RCA, van der Voort A. Rebutting Existing Misconceptions About Multiple Imputation as a Method for Handling Missing Data. J Pers Assess 2020;102:297–308.

[6] Proust-Lima C, Philipps V, Liquet B. Estimation of extended mixed models using latent classes and latent processes: the R package lcmm. J Stat Softw 2017;78. doi:10.18637/jss.v078.i02.

[7] Sabo M, Walker A, Elmi Assadzadeh G, Hildebrand K. Rotator cuff outcomes and mental health indices: Correlation or causation? Shoulder Elb 2022:17585732221076027.

[8] Zou H. The Adaptive Lasso and Its Oracle Properties. J Am Stat Assoc 2006;101:1418–1429.
